# Genomic diversity of Cameroonian Gudali and Gudali-cross cattle

**DOI:** 10.1038/s41598-025-99799-8

**Published:** 2025-04-29

**Authors:** Youchahou Poutougnigni Matenchi, Matthew Hegarty

**Affiliations:** 1 Department of Life Sciences, Aberystwyth University, Penglais Campus, Aberystwyth, Ceredigion SY23 3FL UK; 2 Present Address: Department of Animal Biosciences, Swedish University of Agricultural Sciences, 7023, SE-75007, Uppsala, Sweden

**Keywords:** Animal breeding, Genetic variation

## Abstract

Information on population structure and diversity in cattle breeds is critical for understanding environmental adaptation, as well as optimal utilisation of genetic resources and breed improvement. In this study, we investigated at the genomic level the population structure, genetic diversity and admixture of the local Gudali breed and its crossbred with the Italian Simmental (Simgud) in three agroecological zones of Cameroon. A total of 717 Gudali and 139 Simgud were genotyped using the GeneSeek® Genomic Profiler^TM^ (GGP) Bovine 100K array and analyzed together with reference breed data from public databases. Principal component (PCA) and admixture analysis separated European *Bos taurus* from Asian *Bos indicus*, African *Bos taurus* and African *Bos indicus* breeds. These analyses showed that, except for recently admixed cattle, all African indigenous breeds are either pure African *Bos taurus* (N’dama) or admixtures of African *Bos taurus* and *Bos indicus*. Analysis revealed an ancient admixture from Asian origin in Gudali and a more recent and ongoing European introgression. Simgud is an unmanaged crossbreed expected to be primarily a 50% admixture of Gudali and Simmental. We show here that Simgud is, in actuality, composed of two genetic groups representing admixture of between $\sim$25% to $\sim$50% Simmental proportion. Diversity analysis revealed high average heterozygosity ($Ho=0.34\pm 0.14$, $He=0.35\pm 0.13$) for the Gudali and ($Ho=0.42\pm 0.13$ , $He=0.40\pm 0.11$) for Simgud respectively. Inbreeding measures based on the mean F_IS_ coefficient were 0.03 for Gudali and 0.07 for Simgud. A general decline in effective population size was observed in Gudali from a large population (N_e_=2475), 959 generations (4797 years ago), back to 13 generations (65 years) (N_e_=1404) ago. These results were expected, given the breeding efforts that began in 1952 with the introduction of various exotic (imported taurine) breeds and the Gudali selection initiative. This has affected the effective population size of Gudali, despite the general increase in cattle population in the ranches over that period. These results highlight the need for a structured breeding program in Cameroon for improving productivity, while maintaining a large genetic base of the pure Gudali population.

## Introduction

Several introduction and migration movements have resulted in today’s indigenous African cattle possessing an admixed genome of different proportions - across populations and geographies - of both taurine and zebu ancestries^1–3^. The initial introduction was from domestication center(s) in the Near East which brought taurine cattle into Africa about 7000 years ago^4^. It is nowadays widely accepted - based on evidence such as zooarchaeological remains, Saharan rock art and Egyptian dynastic representations^5^ - that the North East of today’s Egypt^6–8^ was the entry point. The taurine cattle might have been introgressed here with the African wild aurochs *B. primigenius africanus*^3^. From its native South Asia, in the Indus Valley, the zebuine-type cattle entered Africa around 2,000 AD through the Horn of Africa^8^. Later on, the Arab traders’ settlements (about 7th century AD) along the eastern coast of Africa marked the more important wave of zebu introgression^4^. Moreover, the rise of the Swahili civilization, the rinderpest epidemic^4,9,10^ that devastated susceptible African taurine, as well as the environmental adaptation of animals with predominantly indicine background to the dry, hot climate^3,4^, facilitated its wide and fast dispersal towards South and West Africa.

The long-distance westward displacement of the zebu cattle with the Fulani pastoralist ethnic group^4^ put a strong selective pressure on the genome of these animals^11^ to tolerate major diseases, drought, thermal stress, parasites, poor infested pastures and poor management conditions^12^. The remarkable adaptive aptitude developed by the zebu is shown by its wide distribution in different ecological zones of Western and Central Africa^2^. However, this adaptation is often negatively correlated with production performance. In fact, local breeds are well adapted to harsh environmental conditions but produce less than “exotic” taurine breeds highly selected for production performance traits, which conversely are highly susceptible to environmental challenges such as heat stress^13–15^. Replacement or crossbreeding initiatives^4^ of the adapted indigenous zebu^16^ with imported, highly productive European taurines (that are generally bred/reared in controlled facilities^17^) resulted in poorer performance than the indigenous cattle in traditional management systems, where they are diffused to improve production. Moreover, in the absence of proper management schemes, there is also uncontrolled gene flow between breeds with a high risk of loss of genes of interest, the spread of undesired alleles and loss of diversity through genetic uniformity^18^. These recent initiatives, combined with ancient admixture (dating back up to circa 750–1050 yr ago) and the pastoral nature of cattle husbandry in Africa, have resulted in a mosaic of admixed breeds of zebu, taurine and crossbreeds in the African cattle populations^19^.

Cameroon has been engaged in a profound transformation^20^ of its livestock production system to cope with the emerging challenges related to climate, urbanization (with correlated increases in demand for animal products), food insecurity and poverty. Gudali, also known as Peulh or Fulbe zebu, is a West and Central African shorthorn zebu similar in conformation, size, and origin to the East African shorthorn zebu. It is the most popular local breed of Cameroon, especially among small farmers in the Adamawa plateau^21^ and represents about $\sim$60% of the total cattle production in Cameroon^22^. Gudali is a non-specialized breed used commonly as a dual-purpose animal, providing milk and meat^23,24^.It is a well-tempered animal endowed with good adaptation to poor management/harsh environment, produces quite well under low input systems^25^ and thrives optimally under Cameroon’s disease-loaded agroecological conditions. The Gudali breed, because of its well-known production potential, has always been at the center of cattle improvement initiatives in Cameroon. These initiatives started in 1952 and the cattle imported were mainly of Western taurine genetic background including Holstein, Brahman, Salers, Montbeliard, Simmental to combine adaptation aptitude of local breed and production performance of these exotic taurine in a tropical environment^26–29^. Besides these crossbreeding initiatives, the first improvement initiative of the Gudali was launched in 1969 and geared towards improvement of the Gudali breed in the Adamawa region^25^ through selection without crossbreeding. Evaluation of these programmes^30^ pointed to a possible genetic improvement of milk and meat production in Gudali which was even ranked as the best dam breed for production and reproduction traits improvement in Cameroon through crossbreeding with exotic taurine breeds^31^. More recently (2008), the Cameroon National Livestock Company (SODEPA), in its mission to disseminate higher performing and environmentally friendly animals in rural zones, introduced the Italian Simmental via crossbreeding with the local Gudali to produce a hybrid animal named Simgud^29^. It is not yet a stable hybrid and record-keeping is also sparse, limiting the ability to match optimal breed composition to the different agroecology of Cameroon.

These improvement initiatives have been performed without a deep understanding of the genetics underlying adaptation of the Gudali to the various ecological zones, with the risk of affecting the adaptive character and disease resistance of this local resource. A sustainable improvement scheme requires a deep understanding of the genetic background of the local breeds. Outstanding progress has been made in genomic and population genetics with the availability of single nucleotide polymorphism (SNP) arrays and next-generation sequencing (NGS) platforms, offering unprecedented opportunities in cattle genomic and studies are focusing on investigating genetic structure^32^, evaluating genomic diversity^19,33–35^,elucidating the effects of admixture and introgression on cattle breeding in various environments^2,19,36–40^ and breeding schemes are beginning to include heritable adaptation biomarkers in cattle improvement programs^11^. However, to the extent of our knowledge, there is no genome-wide analysis of local zebu breeds - especially Gudali - in terms of admixture and introgression as well as signature of selection. Very few studies have been conducted in Cameroon’s local breeds and these were mainly focused on maternal lineage assessment^41^, microsatellites^18,42^, blood and milk protein markers^18^ and, more recently, a whole genome analysis on a single animal per breed, for their adaptive and disease resistance traits^43^. Genome-wide population studies would help in understanding the genetic structure and genomic composition of the breed - deciphering the adaptive mechanisms, assessing genetic fitness and informing the design of improvement programs. In the present study, we therefore focused on analysing genetic diversity, admixture and introgression, to better understand the genetic basis of local adaptation and functional characteristics of Gudali and Simgud, reared under the same management system in Cameroon.

## Results

### SNP polymorphism

The post-quality filtered dataset consisted of 77,242 variants with a genotyping call rate of 0.99 in 716 Gudali and 139 Simgud cattle. Out of this SNP set, 5412 were haploid genotypes (from the Y chromosome and mitochondrial DNA) and thus removed for the purposes of the genomic variability assessment. The genetic diversity within the Gudali and Simgud populations and their deviation from HWE are shown in Table 1. Percentages of 10% and 0.15% of SNP markers showed significant deviation from HWE (*p*< 0.05) in Gudali and Simgud, respectively. Diversity analysis revealed high average heterozygosity in the Gudali population, with observed heterozygosity (H_o_) of $0.34 \pm 0.14$ and average expected heterozygosity (H_e_) of $0.35 \pm 0.13$. For the Simgud population, the diversity is higher, with the average observed heterozygosity $(0.42 \pm 0.13)$ higher than the expected heterozygosity $(0.40 \pm 0.11)$. Moreover, the Simgud population showed higher levels of inbreeding (0.07) compared to 0.03 for the Gudali population, based on the F_IS_ coefficient.

**Table 1:** Genomic estimates of heterozygosity, inbreeding coefficient and proportion of markers in Hardy-Weinburg Equilibrium in the Gudali and Simgud populations.

Breed	N	He	Ho	F_IS_	HWE
Gudali	717	0.35	0.34	0.03	0.90
Simgud	139	0.41	0.44	0.07	0.85

### Population structure assessment

**Fig. 1:**
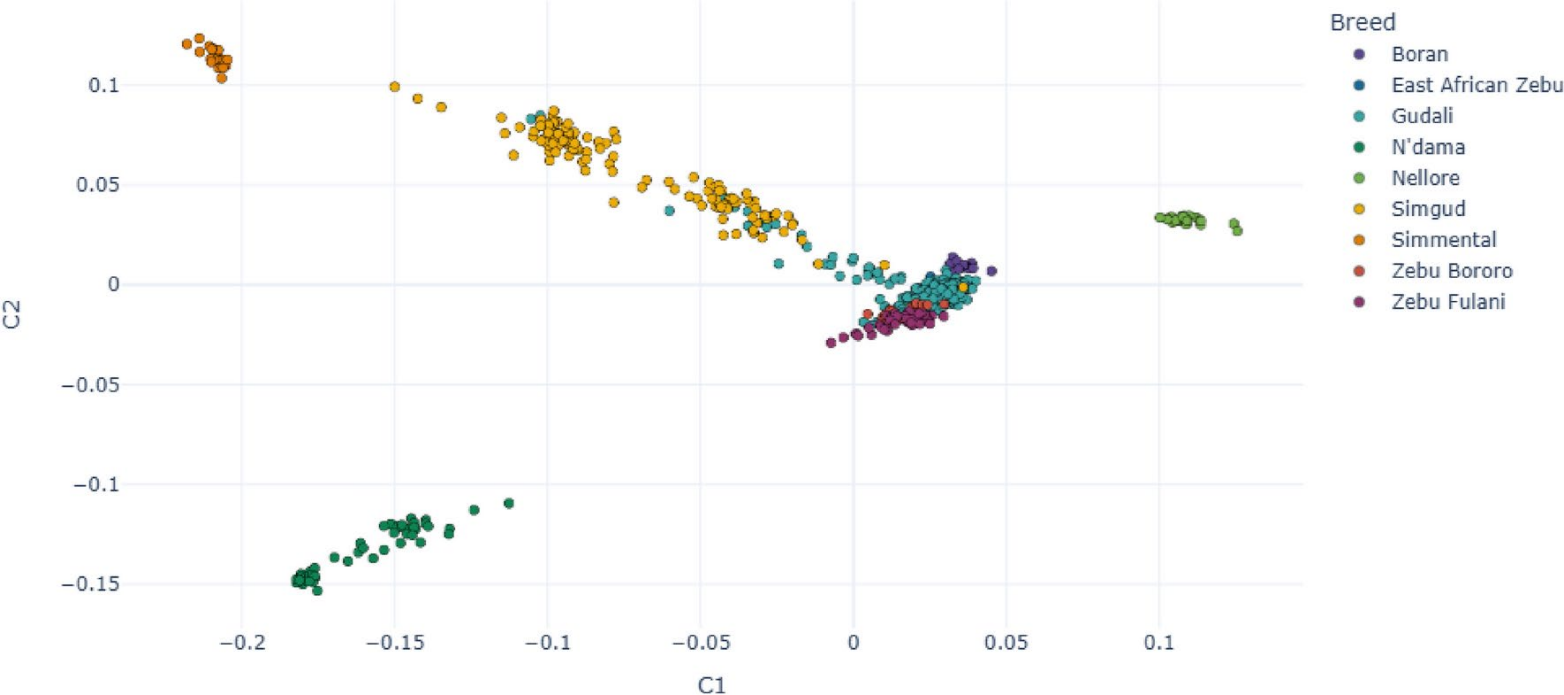
Principal Component Analysis of Gudali and Simgud alongside reference populations: PC1 and PC2 explain 9.04% and 4.2% of variation, respectively.

**Fig. 2:**
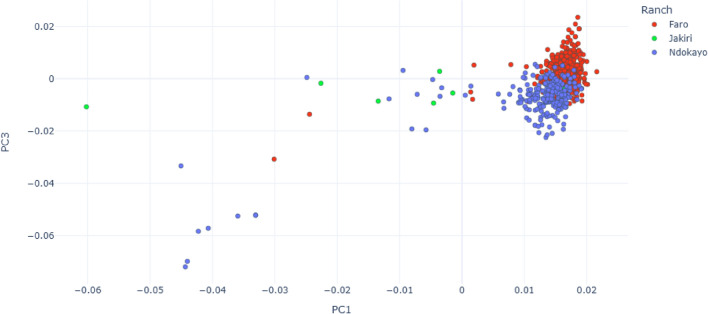
Principal Component Analysis for the Gudali population. PC1 and PC3 explain 1.06% and 0.72% of the variation, respectively.

The PCA analysis shows a clear separation between European taurine (Simmental), Asian zebu (Nellore), African zebu (Boran, East African Zebu, Zebu Bororo, Gudali) and African taurine breed (N’Dama), as shown in Fig. 1. Simgud forms two groupings at $\sim$25% and $\sim$50% between Gudali and Simmental. Considering the Gudali population only, there is a separation on PC1/PC3 between animals from the different ranches (Fig. 2), possibly representing different ecotypes of Gudali.

### Admixture analysis

**Fig. 3:**
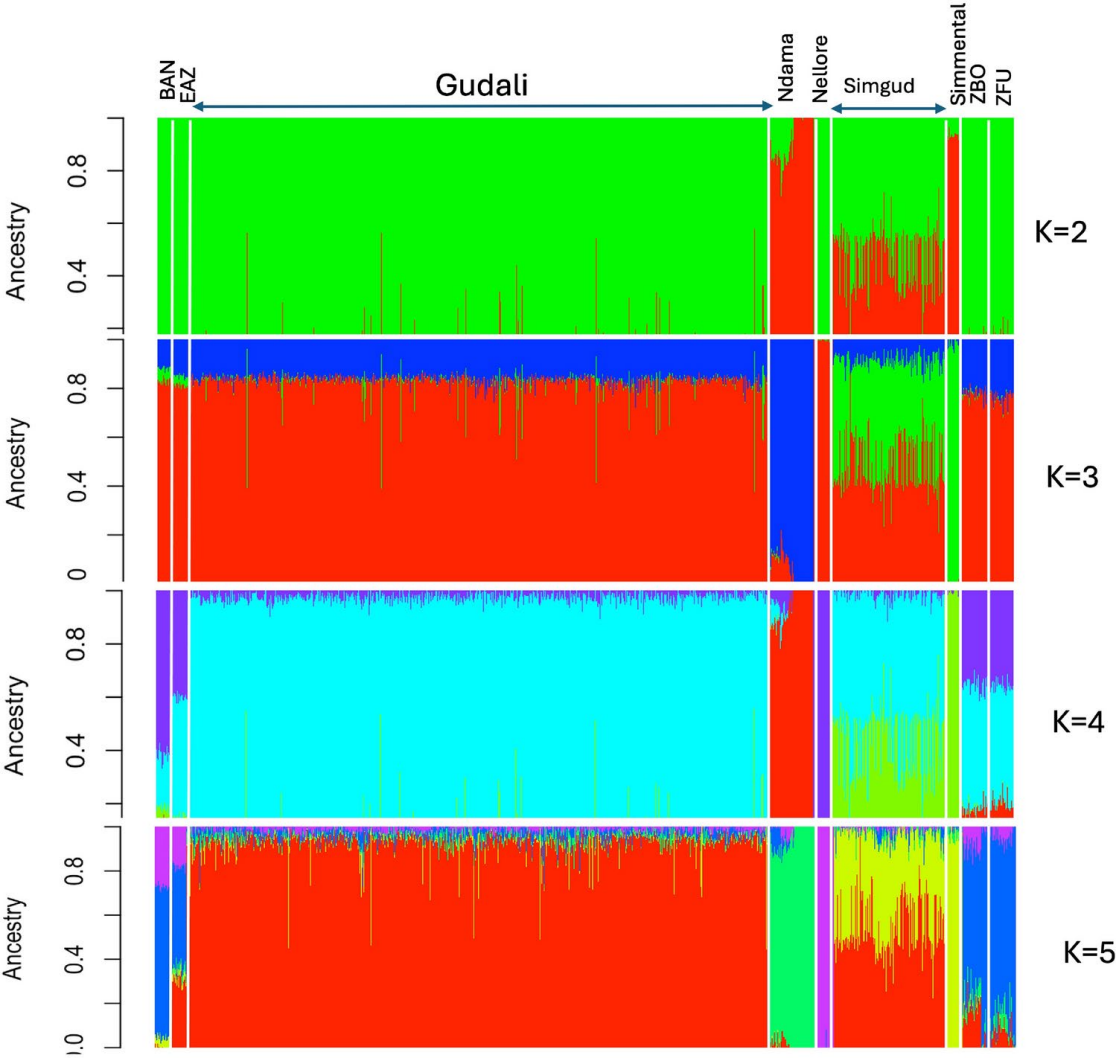
Admixture bar plots of genetic proportions in each animal by breed represented by a vertical line divided into K colours for K=2 to K=5. BAN = Boran, EAZ = East African Zebu, ZBO = Zebu Bororo, ZFU = Zebu Fulani.

**Fig. 4:**
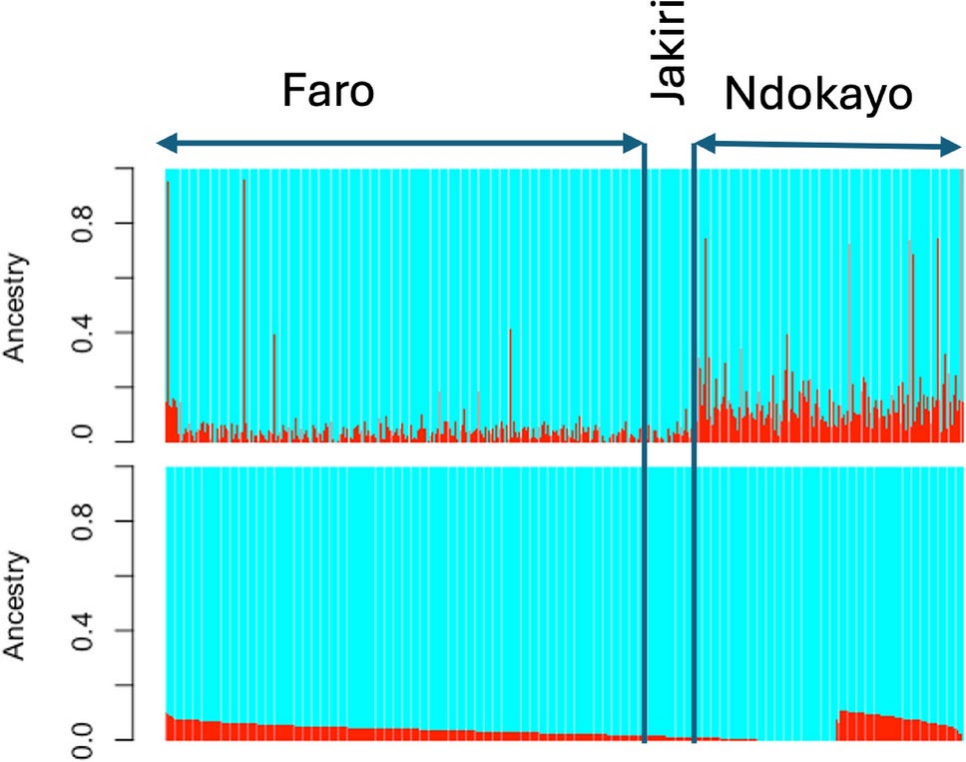
Admixture bar plots of genetic proportions in each Gudali animal by ranch represented by a vertical line divided into K=2 colours, in total Gudali (**A**) and after excluding highly exotic proportion animals (**B**).

Figure 3 shows the estimated breed proportions of the Gudali and Simgud animals from admixture analysis considering European Simmental, Asian Nellore and African indicine (Boran, East African Zebu, Gudali, Zebu Bororo and Zebu Fulani) and African taurine (N’Dama) reference breeds as a potential ancestral breed. We ran the Admixture analysis from K values of 1 to 10. Based on cross-validation (CV) error (see Supplementary Fig. S2), there is no evidence of an “optimal” K, as the CV error seems to decrease minutely with each increasing value of K past 5. However, from visual inspection, K=5 appears to be a reasonable choice, as it is at that point where the decrease in CV error starts to plateau and adding additional parameters does not improve the fit. At K=2, admixture shows a clear separation between taurine (represented by European Simmental and African N’Dama) and indicine population (represented by Asian Nellore and African Boran, East African Zebu, Gudali, Zebu Bororo and Zebu Fulani). Simgud is an admixed population showing introgression of the taurine Simmental component varying between 7% to 74%, with an average of 47%. Increasing K to 3 leads to a finer resolution in the fit of the model which further split the taurine into European (Simmental) and African (N’Dama). K=3 potentially shows a stable ancient African taurine background common to all the African breeds as represented by the blue color, followed by a relatively recent European taurine introgression, more pronounced in East African Boran and East African Zebu breeds. There is a little variation in terms of indicine and African taurine background in all the African breed as opposed to the European taurine background which seems more pronounced in East Africa and only present as trace in West African indicine. There is no further genetic cluster or a new individual breed cluster observed by increasing K to 4. However, increasing K to 5 resulted in a finer resolution in the fit of the model and no further cluster was observed beyond by increasing the goodness of fit with larger K. For the Gudali population only, after excluding animals with more than 10% exotic proportion, the remaining animals (539) - considered as non-introgressed Gudali - were used to rerun the admixture and the result showed a stable Gudali population (Fig. 4).

### Population differentiation analysis

**Fig. 5:**
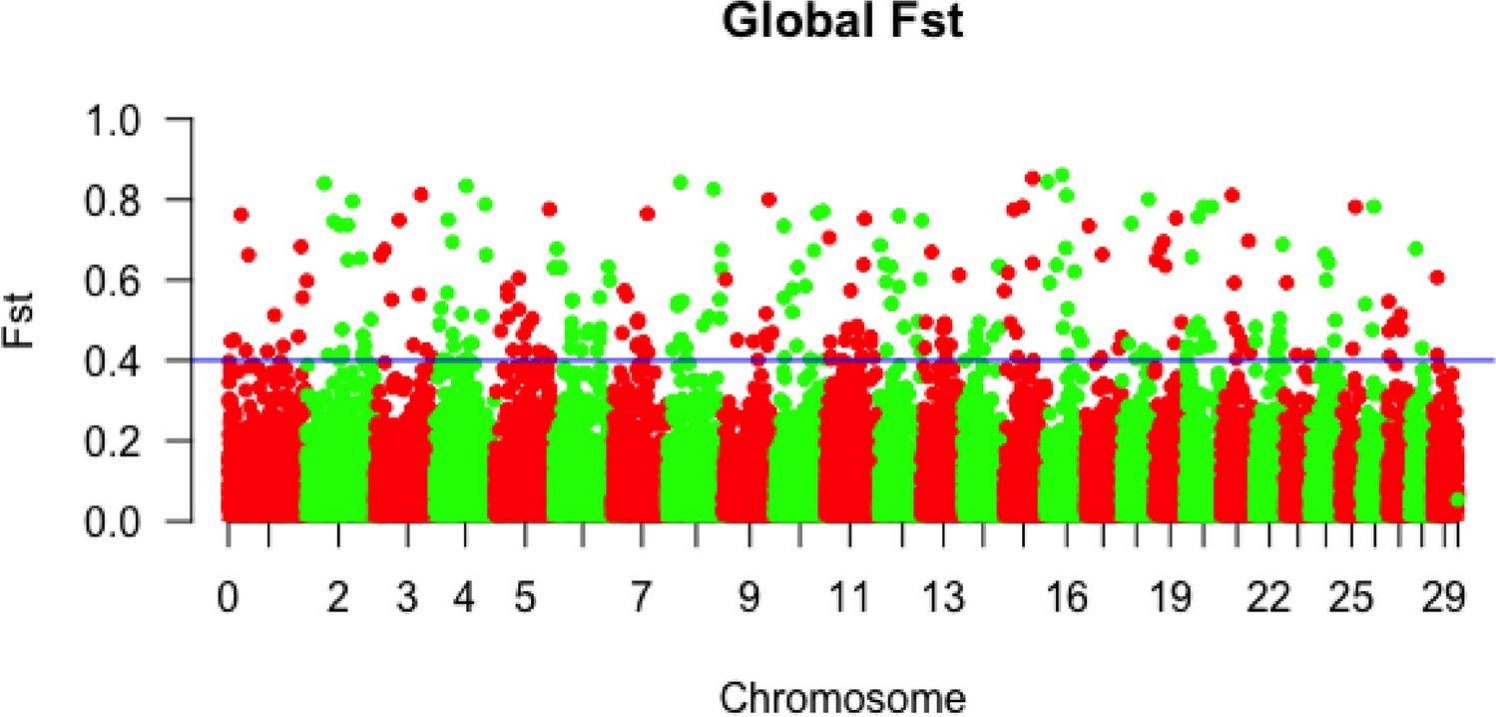
Global distribution of marker F_ST_ values across the chromosomes.

**Fig. 6:**
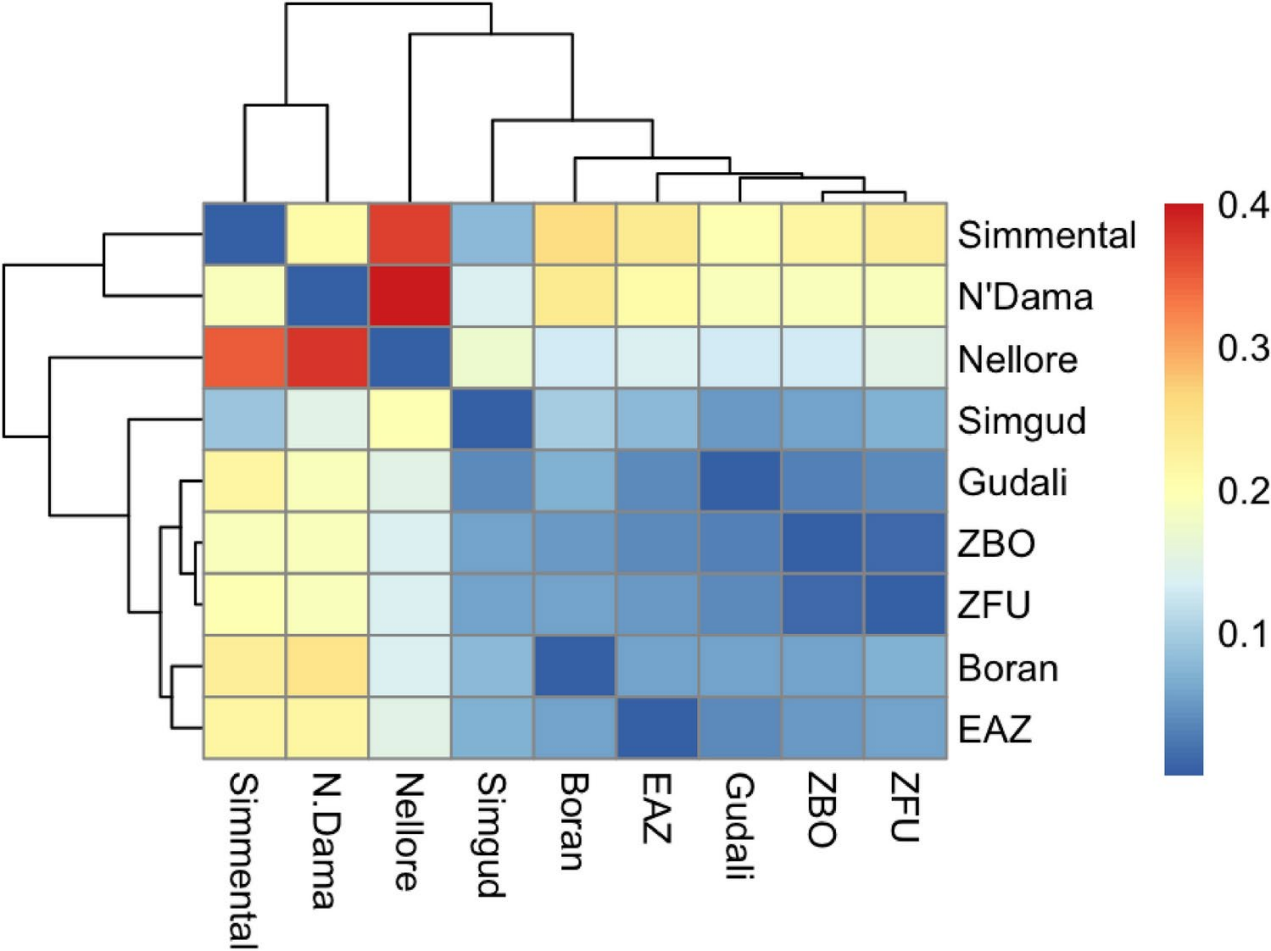
Heatmap of genetic distance between breeds. ZBO = Zebu Bororo, ZFU = Zebu Fulani, EAZ = East African Zebu.

The global marker F_ST_ was computed and its distribution is depicted in Fig. 5. The distribution of F_ST_ showed a considerable number of loci (289 markers) having a high degree of genetic differentiation (F_ST_ $>=$ 0.40). The F_ST_ of all markers showed a mean value of 0.096, falling within the level of moderate genetic differentiation (F_ST_ ranged from 0.05 to 0.15) following Wright’s classification system^44^. Eleven SNPs had an F_ST_ value greater than 0.8, 28 SNPs an F_ST_ between 0.7-0.8, 44 SNPs had an F_ST_ between 0.6 and 0.7, and 50 SNPs had F_ST_ between 0.5 and 0.6. Peaks were observed on BTA2, 3, 4, 8, 15, 16, 18 and 21. The result of the population differentiation analysis performed between Gudali, Simgud and the reference breeds using the pairwise estimates of F-statistics (F_ST_) and Nei’s genetic distance is shown in Table 2 and reinforced by the heatmap of the relationship that shows a separation between taurine and indicine breeds (Fig. 6). The F_ST_ values below 0.05 are considered low differentiation, while those above are considered high following^44^. The highest differentiation was observed between taurine N’Dama and the other breeds, varying from 0.15 to 0.38, followed by taurine Simmental and the remaining breeds, with F_ST_ between 0.09 and 0.35.

Within Gudali, differentiation was performed between locations. The F$_{\textrm{ST}}$ values were very low (mean pairwise F$_{\textrm{ST}} = 0.01$ between Faro and Jakiri, 0.003 between Faro and Ndokayo, 0.009 between Ndokayo and Jakiri). For the differentiation between Gudali and Simgud, we used the full set of filtered markers (77,242 SNPs) and the result gave an F_ST_ of 0.05. Since Simgud is a crossbred of between 25% and 50% of Gudali type, as revealed by the PCA and admixture analysis, a threshold of ${F_{ST}}>=0.2$ would be enough to identify the SNP with greater degree of differentiation. With that threshold, 5,113 SNPs had a high differentiation power between Gudali and Simgud, meaning that 6% of SNP were highly differentiated between Gudali and Simgud. Within Gudali and between Gudali and Simgud F_ST_ estimations are presented in Table 3.

**Table 2:** Pair-wise Nei’s genetic distance (above diagonal) and Weir and Cockerham F_ST_ (below diagonal) among the cattle breeds. EAZ = East African Zebu, ZBO = Zebu Boran, ZFU = Zebu Fulani.

	Gudali	Simgud	Simmental	Boran	EAZ	N’Dama	Nellore	ZBO	ZFU
Gudali	–	0.04	0.22	0.07	0.04	0.19	0.15	0.03	0.04
Simgud	0.05	–	0.09	0.10	0.08	0.15	0.20	0.06	0.07
Simmental	0.20	0.08	–	0.26	0.24	0.21	0.37	0.22	0.23
Boran	0.06	0.08	0.23	–	0.06	0.25	0.14	0.06	0.07
EAZ	0.04	0.07	0.22	0.06	–	0.22	0.15	0.05	0.06
N’Dama	0.19	0.14	0.19	0.24	0.21	–	0.40	0.19	0.19
Nellore	0.13	0.17	0.35	0.13	0.14	0.38	–	0.13	0.15
ZBO	0.03	0.06	0.19	0.05	0.04	0.19	0.14	–	0.01
ZFU	0.04	0.06	0.20	0.06	0.05	0.19	0.14	0.01	–

**Table 3:** Weir and Cockerham F_ST_ within Gudali between locations and between Gudali and Simgud populations.

	Faro	Jakiri	Ndokayo	Gudali
Faro	–			
Jakiri	0.011	–		
Ndokayo	0.003	0.009	–	
Simgud	–	–	–	0.050

**Fig. 7:**
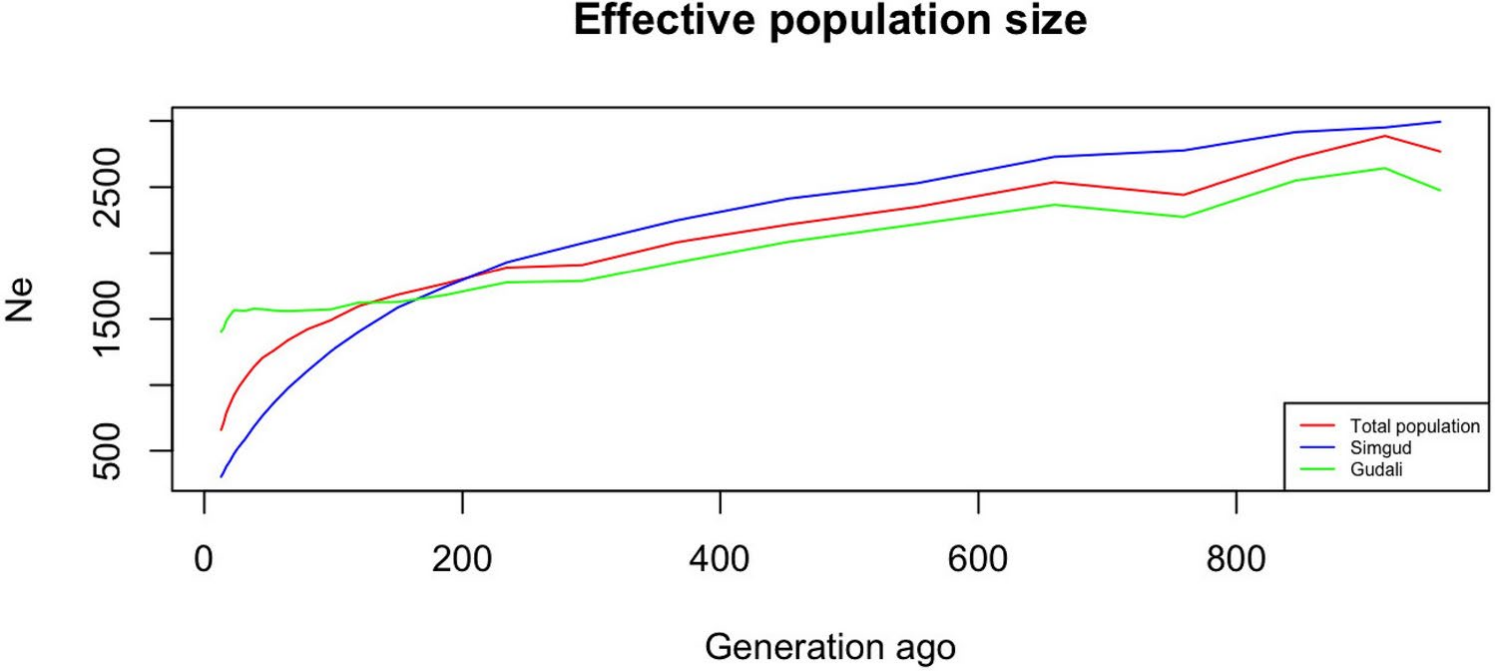
Effective population size in Gudali, Simgud and after introduction of Simgud.

### Effective population size

Results from estimation of the effective population size starting from 13 generations back to 959 generations (selected by the SNeP software as described in Methods) showed that, in the Gudali and Simgud populations, recent generations have similar N_e_ to the surveyed population. When we perform the N_e_ on the Gudali subpopulation, N_e_ was double the number of actual individuals tested, compared to the one obtained using both Gudali and Simgud (Fig. 7).

## Discussion

Our study represents the first attempt to study the diversity of a local cattle breed of Cameroon and its crossbreed with European taurine, using a high-density SNP array. Using H_e_ and H_o_ to measure the genetic diversity, we found moderate values. This result demonstrates that inbreeding affects many loci in the population in general. In both populations, the frequency of heterozygote individuals does not deviate much from 0.5, and indicates that the observed genetic variation corresponds with the expectations, according to Hardy-Weinberg Equilibrium (HWE). Genetic diversity was higher in Simgud than in Gudali, most likely simply because these are crossbred/admixed cattle. Also, Simgud is an unmanaged population that is not registered as a breed. In the absence of pedigree and record keeping, there is no performance evaluation to inform breeding schemes, using high-impact bulls for the next generation. The Simgud population is thus less intensely selected and animals do not share common elite parents. Moreover, the higher diversity observed in Simgud than in Gudali might result from the ascertainment bias of the SNP chip toward European *Bos taurus* during its design^45^. In the Simgud population, H_o_ higher than H_e_ was expected because of the gene flow resulting from introduction of exotic animals into the population. Also, having high genetic diversity appears as a mechanism of adaptation^46^ that might arise from the need to adapt to changing environmental conditions where animals are seeking grazing land during seasons. The same observation about high genetic diversity as result of adaptation to a complex environment was made regarding a large dataset of 34 Chinese cattle breeds by^47^. The H_o_ in Gudali (0.34) in our study coincides with the results from other breeds adapted to challenging environments. The same average H_o_ estimate (0.35) was found in 6 Indian local cattle breeds^40^. Similarly, on nine *Bos indicus* breeds of Brazil, Campos et al.^48^ reported H_o_ estimates varying from 0.32 to 0.39. in Angus and Hanwoo,^49^ found H_o_ values of 0.30 and 0.31 respectively. Likewise, similar H_e_ and H_o_ were observed in Ethiopian indigenous cattle^50^. In Iraqi local cattle breeds^51^ found higher H_e_ (0.37) than in our results. The diversity in Gudali and Simgud is however higher than observed in South African cattle breeds that have H_e_ varying between 0.24 and 0.30^52^ and also showed low inbreeding compared to our results. The considerable genetic diversity observed in our Gudali and Simgud populations, offers an opportunity for setting genetic improvement initiatives to facilitate adaptation of animals to complex local production marked by continued changes in climatic conditions, farm management and food resource availability^53^.

The optimization of any crossbreeding program relies on a deep understanding of the link between genetic admixture and phenotypic performance. In the context of lacking pedigree and record keeping in Cameroon, we performed population structure and admixture analysis of Gudali, a well-known and widely represented West African zebu. The results from the PCA analysis demonstrate the existence of two genetic backgrounds in the Simgud. Some Gudali are distributed in the two Simgud subgroups. Likewise, some Simgud clustered together with Gudali. This observation means that a proportion of animals that were designated as pure Gudali based on phenotypic observation, appear to be crossbred of Gudali and Simmental and a proportion of animals considered to be Simgud crossbred were instead nearly pure indigenous Gudali. Although identification error might cause such observations, similar situations encountered in East African cattle breeds^54^, Indian indigenous cattle breeds^55^, and Australian sheep breeds^56^, indicate that it is not always accurate to determine breed purity based on phenotypic features. The PCA was able to differentiate the groups with the largest genetic differences, i.e. the European taurine, Asian zebu Nellore, other African zebu and taurine breeds. The first two principal components were able to differentiate the groups showing the greatest divergence i.e. PC1 *Bos taurus* vs. *Bos indicus*, PC2 European *Bos taurus* vs. African *Bos taurus*). It also separates close groups such as the African zebu breeds. The ability of PCA to separate between groups with largest genetic differences was also illustrated in previous studies^36,57–60^.

The same tendency was observed with the admixture analysis. At K=2, the admixture showed the divergence between taurine and indicine, indicating the uppermost separation at the subspecies level between taurine and zebu breeds after domestication in the Near Crescent. *Bos taurus* and *Bos indicus*, separated some 200,000 to 300,000 years BP^28,36,59,60^, 575,000 to 800,000 years BP^61,62^ or even 2 M years BP according to^63^. The split between European taurine (Simmental) and African taurine (N’Dama) observed at K=3 matches previously documented findings^32^. This divergence occurred between 180,000 and 250,000 years ago^64^. Mitochondrial DNA (mtDNA) maternal lineage data estimates this separation to be between 22,000 and 26,000 years ago^61^. The African taurine (N’Dama) belongs to the group of humpless cattle that seem to be the earliest cattle domesticated in Africa^8^. These were domesticated by hunter-gatherers around 8000-10,000 years BP^65^, alternatively, in the Grey Crescent and later on crossed with the wild aurochs in Southern Crescent or North Africa - as evidenced genetically by the single domestication event in the Fertile Crescent^57^. The N’Dama population used in this study is a merged group of N’Dama1 (from Cote d’Ivoire), N’Dama2 (from Southeast Burkina Faso), N’Dama3 (from Southwest Burkina Faso) as mentioned in PCA analysis. The N’Dama from Burkina Faso may have included animals of zebu origin^58^. Gudali clustered together with the other Eastern and Western African zebu breeds as expected, all of which show potential signs of a stable admixture as opposed to European taurine introgression which is potentially recent and ongoing. This observation can be explained by the fact that the indicine and African taurine genome ancestries have had enough time to spread homogenously among the breeds under study, while the European taurine introgression is of more recent origin^2^ and has not yet reached a balance. Our study points once again to an admixed genome of all the African indicine groups, confirming the findings from almost all studies so far on this group. These animals may have formed hybrids between *Bos taurus* and *Bos indicus* when migrating to Africa around 4200 years BP^66^. The European taurine introgression in African breeds is still ongoing^2^ and might have provoked the high susceptibility to infectious diseases in breeds such as the East African Short-horn zebu^67^. It is thus widely accepted that there is almost no pure *Bos indicus* in the African continent^58,68^ and their genome is a mosaic resulting from different degrees of admixture of taurine and indicine lineages^69–71^. Admixture results at K=4 did not lead to further separation of breeds. Gudali seems to have arisen from two ancestral backgrounds, probably resulting from the first separation of zebu and taurine after domestication, as well as a third component representing more recent admixture of European genetics due to backcrossing with Simgud. The ancestral backgrounds are more pronounced in Zebu Bororo and East African Shorthorn Zebu, confirming the origin and history of African zebu breeds and previous findings^2,4^. Increasing K to 5 resulted in a finer resolution and no further cluster was observed beyond by increasing the goodness of fit with larger K.

When the Admixture analysis is carried solely within Gudali, there is a common admixture observed, which is more pronounced in the Ndokayo and Faro ranches. This admixture level in Gudali varying between animals needs to reach an equilibrium because it is potentially of recent origin. In fact, Gudali has been selected and used in the very earliest genetic improvement breeding program in Cameroon because of its good meat and milk production potential^23,24^. The similarity with Zebu Fulani, as seen in the admixture plots, reinforces its meat and milk aptitude because the white Fulani are recognized as having good dairy and meat potential^72^. It was reported by^27^ and^26^ that Gudali has been implicated in crossbreeding for beef production improvement initiatives since 1952 with the formation of the wakwa composite and later (1969) in a selection program^21^. More recently, the ongoing program crossing the Italian Simmental and the Gudali was initiated by the SODEPA supported by Italian Simmental Breeders’ Association (ANAPRI) to improve meat and milk production in the Gudali^29^.

Using both PCA and F_ST_ estimation, a clear divergence between different groups of African zebu breeds (Gudali, Boran, East African Zebu, Zebu Bororo, Zebu Fulani), Asian zebu (Nellore), European (Simmental) and African taurine (N’Dama) was observed. There is a closer proximity between taurine Simmental and N’Dama than between African zebu and Asian Nellore. These estimations were expected because of the population history of taurine and indicine breeds from their domestication to their dispersal. The F_ST_ estimations between African zebu show a high degree of similarity between East African (Boran, East African Zebu) and West African zebu (Gudali, Zebu Bororo, Zebu Fulani) breeds. Similar differentiation was observed in Indian^40^ and South African^52^ breeds. Between both African and European taurine and Asian indicine, there is a high differentiation. Taken together, the moderate differentiation between Gudali and Simgud and the moderate to high heterozygosity observed (0.35 and 0.44, respectively), indicate that these populations have the diverse genetic background necessary to adapt to climatic variability^53^.

In the Gudali and Simgud populations, estimates show that recent generations have similar N_e_ to actual sample numbers, suggesting not much loss of genetic diversity in the population. Estimating N_e_ on the Gudali subpopulation, there are some periods of increment in N_e_ between 915 and 958 generations ago, and between 38 and 65 generations ago. At 13 generations (the most recent measured by the SNeP package), N_e_ was almost double (1404) the number of actual individuals tested (717). This demonstrates that the Gudali population has high genetic diversity, unlike in Simgud, where N_e_ at 13th generation is only 35 while the actual population tested is 139 individuals. When Gudali and Simgud are considered together, N_e_ is less (661) than in Gudali alone. Introducing Simmental has thus had a large effect on the local cattle population, halving the effective population size in less than two decades since their introduction. Considering a generation interval of more than 4 years as generally observed in cattle prior to intensive breeding programs^73^, we assumed here a generation interval of 5 years as in^55^. It is shorter than the 6 and 6.72 year interval estimated for the East African short-horn zebu^2^ and Sudanese Kenana respectively^74^. The number of generations (13-959) generations thus corresponds to 65-4795 years ago. The increment in N_e_ observed around 65 years in Gudali coincides with the setting of various Gudali breeding programs in Cameroon between 1952 and 1970 such as the Gudali selection for pure breeding initiative^26,27^. The recent introduction of Simmental marking the resumption of the crossbreeding program had a drastic effect on the Gudali population. The effective population size decreased considerably in just two decades after the introduction of the program. This is an understandable consequence of using only a few Simmental sires - which probably shared some ancestry since European *Bos taurus* might have been domesticated from a smaller population^58^ and have been under intense selection for decades. Composite breeds can be difficult to manage without rotational backcrossing to both parent breeds to maximize diversity and heterosis. Selective pressures (both natural and induced by breeding programmes) can also reshuffle the proportion of the progenitor breeds in a composite over generations^75–77^, as some genomic regions become more favoured over others^78^. Moreover, continuous breeding among the existing Simgud would also result in the loss of heterosis and an increase in inbreeding. To overcome this possible issue, a representative pure Gudali population should be maintained to minimize introgression.

The findings from our study have substantial implications for animal breeding in Cameroon. In the absence of pedigree information and performance record keeping, the study managed to determine the breed composition of the local Gudali and its crossbred Simgud. The Gudali breed has large genetic diversity and very low inbreeding, thus can be used extensively for the diffusion of animal material in rural areas. However, there is a recent and ongoing introgression calling for the development of a structured breeding scheme to maintain the purity of the breed. Simgud is a composite of different proportions of Simmental and Gudali. A breeding program could be designed to test the performance of each different composition of Simgud in the various agroecological zones of Cameroon. Simgud animals are managed as one single population and artificial selection as practiced by SODEPA now, combined with natural selection would lead to a decrease in heterozygosity. Moreover, the reduced effective population size would also increase inbreeding with time. Setting such a breeding program would permit us to identify the genomic regions with a signature of selection, associated with production traits and environmental adaptation in the composite^78^ and improve our understanding of genetic background of traits of economic importance in Gudali and Simgud.

## Methods

### Ethical approval declarations

The relevant permissions from the Ministry of Scientific Research and Innovation (Research permit N0 000156/MINRESI/B00/C00/C10/C13) and ethical clearance from the University of Buea (Permit number: UB-IACUC No 12/2021) were obtained before sample collection. Moreover, the study complies with the Nagoya protocol on access to genetic resources, their derivatives, and associated traditional knowledge through the authorization number: 00014/MINEPDED/CAN/NP-ABS/ABS-FP of May 09, 2022.

### Sampling zone and study population

The Gudali and Simgud samples were collected in different herds, camps of each of the 3 SODEPA ranches located in 3 different agroecological zones of Cameroon (see Supplementary Fig. S1), selected based on the accessibility of the site and the availability of the animals in that period of the year. Blood was collected in vacuum tubes containing EDTA from the animals’ jugular veins, with a sterile 10 ml syringe. The tubes were labeled with the animal ID as on the animal body or its ear tag and stored during the fieldwork (at 0-8^o^C, where possible) and later after the transfer of samples from Cameroon to Ege University Molecular Biology Laboratory, kept at -20^o^C until DNA extraction. The DNA isolation was carried out using a phenol-chloroform-based extraction method. Genomic DNA was checked for its purity via agarose gel electrophoresis, and quantified on a NanoDrop$^{\textrm{TM}}$ 2000 Spectrophotometer. A total of 856 animals (717 Gudali and 139 Simgud) was used in the study.

### Genotyping and quality control

The samples were genotyped by Neogen Europe Ltd. using their GeneSeek® Genomic Profiler^TM^ (GGP) Bovine 100K array (http://www.neogen.com/geneseek/). Genotype data for Gudali and Simgud animals was mapped to the ARS-UCD1.2 genome build and converted to a Variant Call Format (VCF) file uploaded to the European Variant Archive (https://www.ebi.ac.uk/eva/) as project PRJEB79966 and analysis ERZ24835341. We performed quality control of the dataset (Gudali and Simgud) in PLINK 1.07^79^, with parameters set as: minor allele frequencies (MAF) < 0.05 and maximum SNP missingness < 0.1. Individuals with maximum individual missingness < 0.1 were also removed. A total of 77,242 SNPs and 856 animals with a call rate of 0.99 passed the filter and were considered in subsequent analysis. Additionally, some reference breeds were downloaded from the Web-Interfaced next generation Database for genetic Diversity Exploration (WIDDE) resource^80^ and consisted of 24 Nellore, 20 Simmental, 20 Boran (BAN), 20 East African Short-horn Zebu (EAZ), 56 N’dama, 23 Zebu Bororo (ZBO) and 43 Zebu Fulani (ZFU) all genotyped with the Illumina Bovine SNP50v2 array. The total data for 1062 animals were merged in PLINK, retaining only SNPs common to the considered breeds i.e. 23,278 markers. The merged dataset was used for population structure analysis and specific filtering for some analysis will be mentioned in related sections. Details of the used datasets can be found as Supplementary Table A1.

### Heterozygosity estimation

The genetic diversity within the Gudali and Simgud populations was assessed using average observed (H_o_) and expected heterozygosity (H_e_), inbreeding coefficient and Hardy-Weinberg Equilibrium (HWE). The estimation of the considered genetic parameters was performed in PLINK on the Gudali and Simgud dataset of 856 animals and 77,242 SNPs.

### Population structure assessment

For the population structure assessment, a random subset of Gudali (n=130) was selected to avoid bias that might result from oversampling of Gudali whose population was very high compared to the Simgud in the dataset. On this subset, the reference populations were added for stratification and population structure analysis. PCA was performed on this full dataset to depict the relationship between populations. The analysis was based on the identity-by-state (IBS) matrix and performed using the TASSEL 5.2.13 program^81^ and the resulting vectors plotted using the *plotly express* package in Python.

### Admixture analysis

Admixture analysis was carried out using the ADMIXTURE software v1.3.0^82^, describing each animal as admixed based on hypothetical populations. The number of populations (K) was predefined to vary from 1-10. Cross-validation error was calculated for each value of K. Results were plotted using in R as per the Admixture software manual.

### Genetic relationship estimation and genetic differentiation

To perform the different analyses based on the Bovine GGP 100K array, several in-house scripts were written for data reformatting and handling for downstream analysis using both R and Python. Global F_ST_ was calculated using the Weir and Cockerham unbiased estimator^83^ of the Hierfstat R package^84^, to assess divergence between all the breeds under study. The raw global F_ST_ was ranked from the highest value that depicts the potential signature of selection. Negative F_ST_ values were rounded to zero for biological interpretation reasons^85^. The genome-wide distribution of marker F_ST_ was displayed in the form of a Manhattan plot using the QQman package in R^86^. Between population differentiation analysis was performed between Gudali, Simgud and the reference breeds using Weir and Cockerham’s F_ST_ estimator^83^ (weighted by population sample sizes) and Nei genetic distances^87^ of the Hierfstat package 0.5-11, in R software. The F_ST_ estimator refers to the genetic variability between populations expressed as a proportion of the total genetic variance.

### Effective population size

To assess the demographic history of the cattle populations, the effective population size was estimated starting from 13 generations back to 959 generations ago, using the SNeP v1.1 software^88^ on the Gudali-Simgud population and later on each population separately. The estimation infers N_e_ based on LD against past *t* generations, with $t=1/2c$ and c representing the between SNP distance in Morgans, assuming (100 mbp = 1 Morgan)^89^ and a generation interval of 5 years^55^.

## Supplementary Information

Supplementary Figure S1.

Supplementary Figure S2.

Supplementary Table S1.

## Data Availability

Genotype data for Gudali and Simgud animals is available as a VCF file mapped to the ARS-UCD1.2 genome build and hosted at European Variant Archive (https://www.ebi.ac.uk/eva/) as project PRJEB79966 and analysis ERZ24835341.

## References

[1] Bahbahani, H. et al. Signatures of selection for environmental adaptation and zebu taurine hybrid fitness in East African Shorthorn Zebu. *Front. Genet.* **8**, 68 (2017). 28642786 10.3389/fgene.2017.00068 PMC5462927

[2] Mbole-Kariuki, M. N. *et al.* Genome-wide analysis reveals the ancient and recent admixture history of East African shorthorn zebu from western Kenya (2014). 10.1038/hdy.2014.31 PMC4181064 24736786

[3] Decker, J. E. et al. Worldwide patterns of ancestry, divergence, and admixture in domesticated cattle. *PLoS Genet.* **10**, e1004254 (2014). 24675901 10.1371/journal.pgen.1004254 PMC3967955

[4] Hanotte, O. et al. African pastoralism: genetic imprints of origins and migrations. *Science* **296**, 336–339 (2002). 11951043 10.1126/science.1069878

[5] Gebrehiwot, N. Z., Strucken, E. M., Marshall, K., Aliloo, H. & Gibson, J. P. SNP panels for the estimation of dairy breed proportion and parentage assignment in African crossbred dairy cattle. *Genet. Sel. Evol.* **53**, 1–18 (2021). 33653262 10.1186/s12711-021-00615-4 PMC7923343

[6] Epstein, H. The origin of the domestic animals of Africa. vol. ii. (1971).

[7] Blench, R. & MacDonald, K. The origins and development of African livestock: archaeology, genetics, linguistics and ethnography (2006).

[8] Gifford-Gonzalez, D. & Hanotte, O. Domesticating animals in Africa: Implications of genetic and archaeological findings. *J. World Prehist.* **24**, 1–23 (2011).

[9] Blench, R. Ethnographic and linguistic evidence for the prehistory of African ruminant livestock, horses and ponies 1. In *The Archaeology of Africa*, 71–103 (Routledge, 2014).

[10] Payne, W. J. A. & Hodges, J. Tropical cattle: origins, breeds and breeding policies. (1997).

[11] Ben-Jemaa, S. et al. Whole genome sequencing reveals signals of adaptive admixture in creole cattle. *Sci. Rep.* **13**, 12155 (2023). 37500674 10.1038/s41598-023-38774-7 PMC10374910

[12] Williamson, G. & Payne, W. An introduction to animal husbandry in the tropics. (1978).

[13] Gaughan, J. B., Sejian, V., Mader, T. L. & Dunshea, F. R. Adaptation strategies: ruminants. *Anim. Front.* **9**, 47–53 (2019). 32002239 10.1093/af/vfy029 PMC6951952

[14] Madhusoodan, A. P. et al. Resilient capacity of cattle to environmental challenges-an updated review. *J. Anim. Behav. Biometeorol.* **7**, 104–118 (2019).

[15] Angel, S. et al. Climate change and cattle production-impact and adaptation. *J. Vet. Med. Res.* **5**, 1134 (2018).

[16] Renaudeau, D. et al. Adaptation to hot climate and strategies to alleviate heat stress in livestock production. *Animal* **6**, 707–728 (2012). 22558920 10.1017/S1751731111002448

[17] Mekonnen, T., Tadesse, Y. & Meseret, S. Genetic improvement strategy of indigenous cattle breeds: Effect of cattle crossbreeding program in production performances. *J. Appl. Life Sci. Int.* **23**, 23–40 (2020).

[18] Ibeagha-Awemu, E. & Erhardt, G. Genetic structure and differentiation of 12 African *Bos indicus* and *Bos taurus* cattle breeds, inferred from protein and microsatellite polymorphisms. *J. Anim. Breed. Genet.* **122**, 12–20 (2005). 16130484 10.1111/j.1439-0388.2004.00478.x

[19] Kim, K. et al. The mosaic genome of indigenous African cattle as a unique genetic resource for African pastoralism. *Nat. Genet.* **52**, 1099–1110 (2020). 32989325 10.1038/s41588-020-0694-2

[20] Mwai, O., Hanotte, O., Kwon, Y.-J. & Cho, S. African indigenous cattle: Unique genetic resources in a rapidly changing world. *Asian Australas. J. Anim. Sci.* **28**, 911 (2015). 26104394 10.5713/ajas.15.0002R PMC4478499

[21] Tawah, C., Mbah, D. & Lhoste, P. Effects of bos taurus genes on pre-weaning growth of zebu cattle on the Adamawa highlands, Cameroon. (1996).

[22] Bayemi, P. *et al.* Participatory rural appraisal of dairy farms in the north west province of Cameroon. *Livestock Res. Rural Dev.* **17** (2005).

[23] Djoko, T. *et al.* Crossbreeding cattle for milk production in the tropics: effects of genetic and environmental factors on the performance of improved genotypes on the Cameroon western high plateau= cruces de ganado para la produccion lactea en los tropicos: efectos de los factores geneticos y ambientales en el rendimiento de los genotipos mejorados en las mesetas atlas de camerun del oeste. croisement des bovins pour la production laitiere sous les tropiques: effets des facteurs génétiques et environnementaux sur les performances des genotypes améliorés des hauts-plateaux de l’ouest cameroun (2003).

[24] Kouamo, J., Sopbue, I. K., Hayatou, S. & Zoli, A. P. Evaluation of reproductive and milk production performances of Gudali, Holstein and crossbred cows by morphobiometry on traditional small-scale farms in Ngaoundere, Adamawa region (Cameroon). *Veterinaria* **68** (2019).

[25] Tawah, C. & Rege, J. Gudali cattle of west and central Africa (1996).

[26] Mandon, A. et al. Le zébu brahma au cameroun: premiers résultats de son introduction en adamawa. *Rev. Elev. Med. Vet. Pays Trop.* **10**, 129–145 (1957).

[27] Lhoste, P. Les races bovines de l’adamaoua (cameroun) (1969).

[28] Loftus, R. T., MacHugh, D. E., Bradley, D. G., Sharp, P. M. & Cunningham, P. Evidence for two independent domestications of cattle. *Proc. Natl. Acad. Sci.* **91**, 2757–2761 (1994). 8146187 10.1073/pnas.91.7.2757 PMC43449

[29] Bessong, W. O. Improving bovine productivity in central Africa: The case of Goudali zebu cattle under ranching conditions in western highland Sudan-Savannah of Cameroon (2016).

[30] Ebangi, A., Erasmus, G., Neser, F. & Tawah, C. Genetic trends for growth in the Gudali and Wakwa cattle breeds of Cameroon. *S. Afr. J. Anim. Sci.* **30**, 36–37 (2000).

[31] Djoko, T. et al. Croisement des bovins pour la production laitière sous les tropiques: Effets des facteurs génétiques et environnementaux sur les performances des génotypes améliorés des hauts-plateaux de l’Ouest cameroun. *Rev. Elev. Med. Vet. Pays Trop.* **56**, 63–72 (2003).

[32] Consortium, et al. Genome-wide survey of SNP variation uncovers the genetic structure of cattle breeds. *Science* **324**, 528–532 (2009). 19390050 10.1126/science.1167936 PMC2735092

[33] Gautier, M. et al. A whole genome Bayesian scan for adaptive genetic divergence in West African cattle. *BMC Genomics* **10**, 1–18 (2009). 19930592 10.1186/1471-2164-10-550 PMC2784811

[34] McTavish, E. J. & Hillis, D. M. A genomic approach for distinguishing between recent and ancient admixture as applied to cattle. *J. Hered.* **105**, 445–456 (2014). 24510946 10.1093/jhered/esu001 PMC4048551

[35] Liu, D. et al. Genetic parameters and genome-wide association for milk production traits and somatic cell score in different lactation stages of shanghai holstein population. *Front. Genet.* **13**, 940650 (2022). 36134029 10.3389/fgene.2022.940650 PMC9483179

[36] Chen, N. et al. Whole-genome resequencing reveals world-wide ancestry and adaptive introgression events of domesticated cattle in east asia. *Nat. Commun.* **9**, 2337 (2018). 29904051 10.1038/s41467-018-04737-0 PMC6002414

[37] Barbato, M. et al. Adaptive introgression from indicine cattle into white cattle breeds from central Italy. *Sci. Rep.* **10**, 1279 (2020). 31992729 10.1038/s41598-020-57880-4 PMC6987186

[38] Medugorac, I. et al. Whole-genome analysis of introgressive hybridization and characterization of the bovine legacy of Mongolian yaks. *Nat. Genet.* **49**, 470–475 (2017). 28135247 10.1038/ng.3775

[39] Mukherjee, A. et al. High-density genotyping reveals genomic characterization, population structure and genetic diversity of Indian mithun (*Bos frontalis*). *Sci. Rep.* **8**, 10316 (2018). 29985484 10.1038/s41598-018-28718-x PMC6037757

[40] Dixit, S. et al. Genome analyses revealed genetic admixture and selection signatures in *Bos indicus*. *Sci. Rep.* **11**, 21924 (2021). 34753978 10.1038/s41598-021-01144-2 PMC8578574

[41] Ngono, E., Meutchieye, F., Keambou, T. & Manjeli, Y. Genetic diversity and origin of namchi cattle breed inferred by matrilineage analyses. (2018).

[42] Ema, P. N. et al. Genetic diversity of four Cameroonian indigenous cattle using microsatellite markers. *J. Livestock Sci.* **5**, 9–17 (2014).

[43] Paguem, A. et al. Whole genome characterization of autochthonous *Bos taurus brachyceros* and introduced *Bos indicus indicus* cattle breeds in Cameroon regarding their adaptive phenotypic traits and pathogen resistance. *BMC Genet.* **21**, 1–15 (2020). 32571206 10.1186/s12863-020-00869-9 PMC7309992

[44] Wright, S. Variability within and among natural populations. *Evol. Genet. Popul.* **4** (1984).

[45] Matukumalli, L. K. et al. Development and characterization of a high density SNP genotyping assay for cattle. *PLoS ONE* **4**, e5350 (2009). 19390634 10.1371/journal.pone.0005350 PMC2669730

[46] Trujano-Chavez, M. Z., Ruíz-Flores, A., López-Ordaz, R. & Pérez-Rodríguez, P. Genetic diversity in reproductive traits of Braunvieh cattle determined with SNP markers. *Vet. Med. Sci.* **8**, 1709–1720 (2022). 35545927 10.1002/vms3.836 PMC9297803

[47] Xia, X. et al. Genetic diversity of Chinese cattle revealed by Y-SNP and Y-STR markers. *Anim. Genet.* **50**, 64–69 (2019). 30421442 10.1111/age.12742

[48] Campos, B. M. et al. Genetic diversity, population structure, and correlations between locally adapted zebu and taurine breeds in Brazil using SNP markers. *Trop. Anim. Health Prod.* **49**, 1677–1684 (2017). 28808902 10.1007/s11250-017-1376-7

[49] Nawaz, M. Y., Savegnago, R. P., Lim, D., Lee, S. H. & Gondro, C. Signatures of selection in Angus and Hanwoo beef cattle using imputed whole genome sequence data. *Front. Genet.* **15**, 1368710 (2024). 39161420 10.3389/fgene.2024.1368710 PMC11331311

[50] Edea, Z., Dadi, H., Kim, S. W., Dessie, T. & Kim, K.-S. Comparison of SNP variation and distribution in indigenous Ethiopian and Korean cattle (Hanwoo) populations. *Genomics Inform.* **10**, 200 (2012). 23166531 10.5808/GI.2012.10.3.200 PMC3492656

[51] Alshawi, A., Essa, A., Al-Bayatti, S. & Hanotte, O. Genome analysis reveals genetic admixture and signature of selection for productivity and environmental traits in Iraqi cattle. *Front. Genet.* **10**, 609 (2019). 31379916 10.3389/fgene.2019.00609 PMC6646475

[52] Makina, S. O., Muchadeyi, F. C., van Marle-Köster, E., MacNeil, M. D. & Maiwashe, A. Genetic diversity and population structure among six cattle breeds in South Africa using a whole genome SNP panel. *Front. Genet.* **5**, 333 (2014). 25295053 10.3389/fgene.2014.00333 PMC4170099

[53] Thornton, P. K. Livestock production: Recent trends, future prospects. *Philos. Trans. R. Soc. B Biol. Sci.* **365**, 2853–2867 (2010). 10.1098/rstb.2010.0134 PMC2935116 20713389

[54] Strucken, E. M. et al. Genetic tests for estimating dairy breed proportion and parentage assignment in East African crossbred cattle. *Genet. Sel. Evol.* **49**, 1–18 (2017). 28899355 10.1186/s12711-017-0342-1 PMC5596489

[55] Strucken, E. M. et al. Genetic diversity and effective population sizes of thirteen Indian cattle breeds. *Genet. Sel. Evol.* **53**, 1–17 (2021). 34074236 10.1186/s12711-021-00640-3 PMC8170732

[56] Weerasinghe, W. *The Accuracy and Bias of Estimates of Breed Composition and Inference About Genetic Structure Using High Density SNP Markers in Australian Sheep Breeds* (University of New England, 2014).

[57] Achilli, A. et al. Mitochondrial genomes of extinct aurochs survive in domestic cattle. *Curr. Biol.* **18**, R157–R158 (2008). 18302915 10.1016/j.cub.2008.01.019

[58] Gebrehiwot, N. Z., Strucken, E., Aliloo, H., Marshall, K. & Gibson, J. P. The patterns of admixture, divergence, and ancestry of African cattle populations determined from genome-wide SNP data. *BMC Genomics* **21**, 1–16 (2020). 10.1186/s12864-020-07270-x PMC7720612 33287702

[59] Bradley, D. G., MacHugh, D. E., Cunningham, P. & Loftus, R. T. Mitochondrial diversity and the origins of African and European cattle. *Proc. Natl. Acad. Sci.* **93**, 5131–5135 (1996). 8643540 10.1073/pnas.93.10.5131 PMC39419

[60] Wu, D.-D. et al. Pervasive introgression facilitated domestication and adaptation in the *Bos* species complex. *Nat. Ecol. Evol.* **2**, 1139–1145 (2018). 29784979 10.1038/s41559-018-0562-y

[61] Loftus, R. et al. Mitochondrial genetic variation in European, African and Indian cattle populations. *Anim. Genet.* **25**, 265–271 (1994). 7985843 10.1111/j.1365-2052.1994.tb00203.x

[62] Wang, K. et al. Incomplete lineage sorting rather than hybridization explains the inconsistent phylogeny of the wisent. *Commun. Biol.* **1**, 169 (2018). 30374461 10.1038/s42003-018-0176-6 PMC6195592

[63] Hiendleder, S., Lewalski, H. & Janke, A. Complete mitochondrial genomes of *Bos taurus* and *Bos indicus* provide new insights into intra-species variation, taxonomy and domestication. *Cytogenet. Genome Res.* **120**, 150–156 (2008). 18467841 10.1159/000118756

[64] MacHugh, D. E., Shriver, M. D., Loftus, R. T., Cunningham, P. & Bradley, D. G. Microsatellite DNA variation and the evolution, domestication and phylogeography of taurine and zebu cattle (*Bos taurus* and *bos indicus*). *Genetics* **146**, 1071–1086 (1997). 9215909 10.1093/genetics/146.3.1071 PMC1208036

[65] Marshall, F. & Hildebrand, E. Cattle before crops: the beginnings of food production in Africa. *J. World Prehist.* **16**, 99–143 (2002).

[66] Verdugo, M. P. et al. Ancient cattle genomics, origins, and rapid turnover in the fertile crescent. *Science* **365**, 173–176 (2019). 31296769 10.1126/science.aav1002

[67] Murray, G. G. et al. Genetic susceptibility to infectious disease in East African Shorthorn Zebu: A genome-wide analysis of the effect of heterozygosity and exotic introgression. *BMC Evol. Biol.* **13**, 1–8 (2013). 24209611 10.1186/1471-2148-13-246 PMC3828575

[68] Weerasinghe, S., Gibson, J., Gondro, C. & Jeyaruban, G. Use of genetic polymorphisms to assess the genetic structure and breed composition of crossbred animals (2016).

[69] Kim, J. et al. The genome landscape of indigenous African cattle. *Genome Biol.* **18**, 1–14 (2017). 28219390 10.1186/s13059-017-1153-y PMC5319050

[70] Pitt, D. et al. Domestication of cattle: Two or three events?. *Evol. Appl.* **12**, 123–136 (2019). 30622640 10.1111/eva.12674 PMC6304694

[71] Friedrich, J. et al. Mapping restricted introgression across the genomes of admixed indigenous African cattle breeds. *Genet. Sel. Evol.* **55**, 91 (2023). 38097935 10.1186/s12711-023-00861-8 PMC10722721

[72] Pullan, N. & Grindle, R. Productivity of white Fulani cattle on the Jos plateau, Nigeria. iv. Economic factors. *Trop. Anim. Health Prod.* **12**, 161–170 (1980). 7434477 10.1007/BF02242648

[73] Kidd, K. & Cavalli-Sforza, L. The role of genetic drift in the differentiation of Icelandic and Norwegian cattle. *Evolution* 381–395 (1974). 10.1111/j.1558-5646.1974.tb00759.x 28564858

[74] Alim, K. Reproductive rates and milk yield of Kenana cattle in Sudan. *J. Agric. Sci.* **55**, 183–188 (1960).

[75] Hay, E. H. et al. Genetic architecture of a composite beef cattle population. *J. Anim. Sci.* **100**, skac230 (2022). 35771897 10.1093/jas/skac230 PMC9467035

[76] Paim, T. D. P. et al. Dynamics of genomic architecture during composite breed development in cattle. *Anim. Genet.* **51**, 224–234 (2020). 31961956 10.1111/age.12907 PMC7065137

[77] Paim, T. D. P. et al. Genomic breed composition of selection signatures in Brangus beef cattle. *Front. Genet.* **11**, 710 (2020). 32754198 10.3389/fgene.2020.00710 PMC7365941

[78] Goszczynski, D. E. et al. Evidence of positive selection towards zebuine haplotypes in the Bola region of Brangus cattle. *Animal* **12**, 215–223 (2018). 28707606 10.1017/S1751731117001380

[79] Purcell, S. et al. Plink: A tool set for whole-genome association and population-based linkage analyses. *Am. J. Hum. Genet.* **81**, 559–575 (2007). 17701901 10.1086/519795 PMC1950838

[80] Sempéré, G. et al. Widde: A web-interfaced next generation database for genetic diversity exploration, with a first application in cattle. *BMC Genomics* **16**, 1–8 (2015). 26573482 10.1186/s12864-015-2181-1 PMC4647285

[81] Bradbury, P. et al. TASSEL: Software for association mapping of complex traits in diverse samples. *Bioinformatics* **23**, 2633–2635 (2007). 17586829 10.1093/bioinformatics/btm308

[82] Alexander, D. H., Novembre, J. & Lange, K. Fast model-based estimation of ancestry in unrelated individuals. *Genome Res.* **19**, 1655–1664 (2009). 19648217 10.1101/gr.094052.109 PMC2752134

[83] Weir, B. S. & Cockerham, C. C. Estimating f-statistics for the analysis of population structure. *Evolution* 1358–1370 (1984). 10.1111/j.1558-5646.1984.tb05657.x 28563791

[84] Goudet, J. Hierfstat, a package for r to compute and test hierarchical f-statistics (2005).

[85] Akey, J. M., Zhang, G., Zhang, K., Jin, L. & Shriver, M. D. Interrogating a high-density SNP map for signatures of natural selection. *Genome Res.* **12**, 1805–1814 (2002). 12466284 10.1101/gr.631202 PMC187574

[86] Team & R. C,. R language definition. *Vienna, Austria: R foundation for statistical computing* **3**, 116 (2000).

[87] Nei, M. *Molecular Evolutionary Genetics* (Columbia University Press, 1987).

[88] Barbato, M., Orozco-terWengel, P., Tapio, M. & Bruford, M. W. Snep: A tool to estimate trends in recent effective population size trajectories using genome-wide SNP data. *Front. Genet.* **6**, 109 (2015). 25852748 10.3389/fgene.2015.00109 PMC4367434

[89] Barbato, M. et al. Genomic signatures of adaptive introgression from European mouflon into domestic sheep. *Sci. Rep.* **7**, 7623 (2017). 28790322 10.1038/s41598-017-07382-7 PMC5548776

