# Supplementary figures and images for: Genomic diversity of Cameroonian Gudali and Gudali-cross cattle
Source: Sci Rep. 2025 Apr 29;15:15066. doi: 10.1038/s41598-025-99799-8 (PMC12041558; doi:10.1038/s41598-025-99799-8)

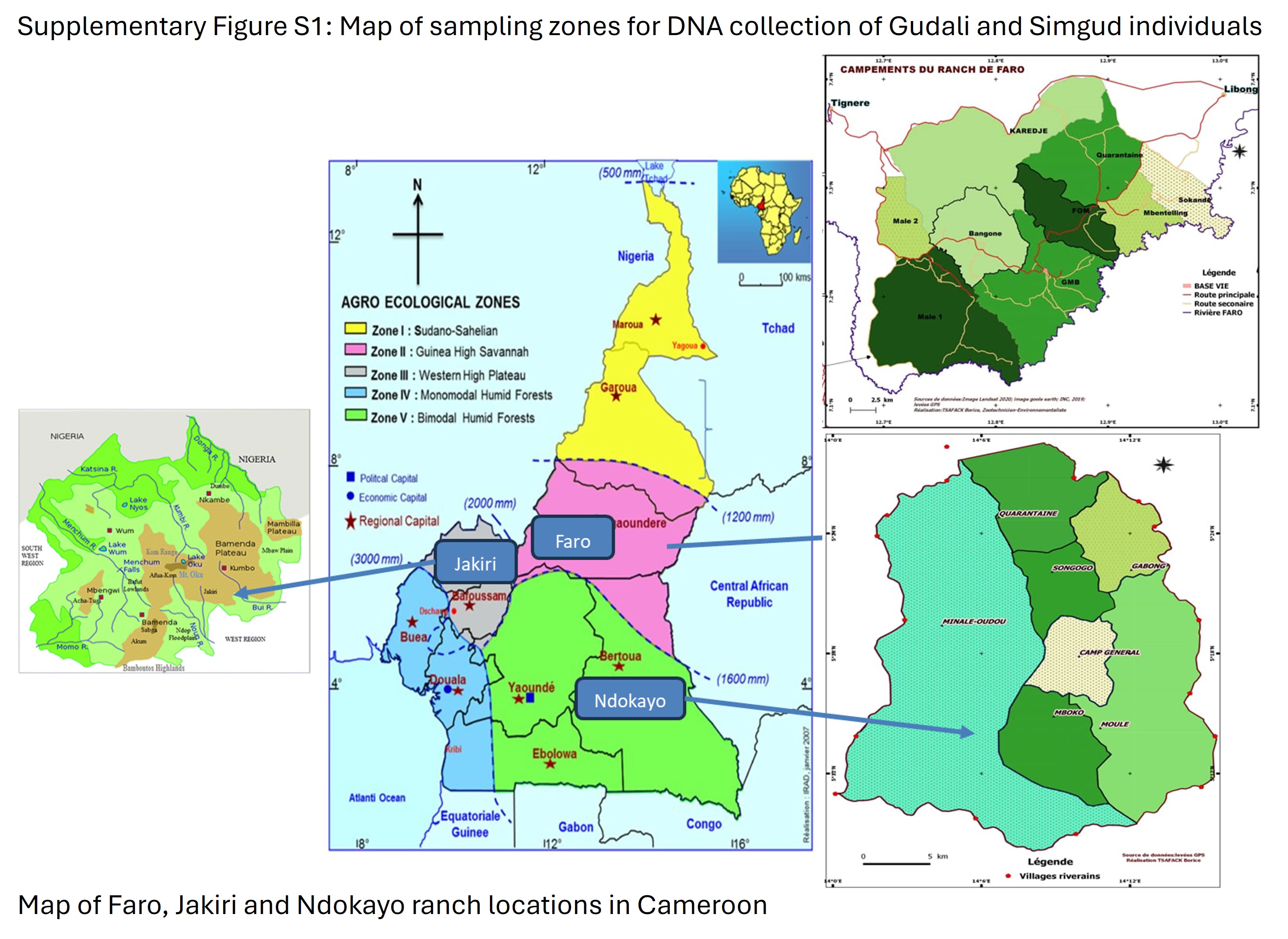

Supplement: Supplementary file 1 — Supplementary Figure S1. [file 41598_2025_99799_MOESM1_ESM.jpg]

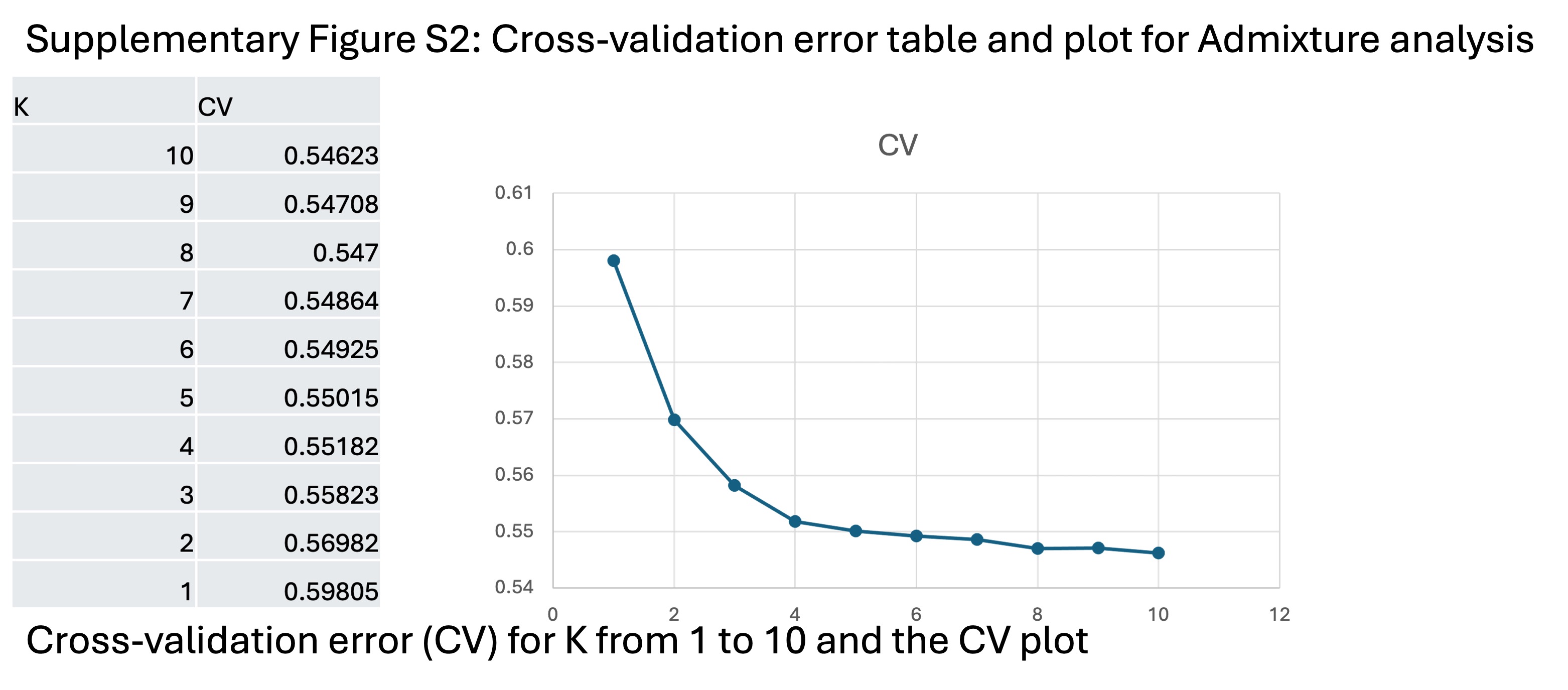

Supplement: Supplementary file 2 — Supplementary Figure S2. [file 41598_2025_99799_MOESM2_ESM.jpg]
